# 3DMMS: robust 3D Membrane Morphological Segmentation of C. elegans embryo

**DOI:** 10.1186/s12859-019-2720-x

**Published:** 2019-04-08

**Authors:** Jianfeng Cao, Ming-Kin Wong, Zhongying Zhao, Hong Yan

**Affiliations:** 1 Department of Electronic Engineering, City University of Hong Kong, Kowloon Tong, Hong Kong; 2 Department of Biology, Hong Kong Baptist University, Kowloon Tong, Hong Kong

**Keywords:** 3D morphological segmentation, Watershed segmentation, Shape features, *C. elegans*

## Abstract

**Background:**

Understanding the cellular architecture is a fundamental problem in various biological studies. *C. elegans* is widely used as a model organism in these studies because of its unique fate determinations. In recent years, researchers have worked extensively on *C. elegans* to excavate the regulations of genes and proteins on cell mobility and communication. Although various algorithms have been proposed to analyze nucleus, cell shape features are not yet well recorded. This paper proposes a method to systematically analyze three-dimensional morphological cellular features.

**Results:**

Three-dimensional Membrane Morphological Segmentation (3DMMS) makes use of several novel techniques, such as statistical intensity normalization, and region filters, to pre-process the cell images. We then segment membrane stacks based on watershed algorithms. 3DMMS achieves high robustness and precision over different time points (development stages). It is compared with two state-of-the-art algorithms, RACE and BCOMS. Quantitative analysis shows 3DMMS performs best with the average Dice ratio of 97.7% at six time points. In addition, 3DMMS also provides time series of internal and external shape features of *C. elegans*.

**Conclusion:**

We have developed the 3DMMS based technique for embryonic shape reconstruction at the single-cell level. With cells accurately segmented, 3DMMS makes it possible to study cellular shapes and bridge morphological features and biological expression in embryo research.

**Electronic supplementary material:**

The online version of this article (10.1186/s12859-019-2720-x) contains supplementary material, which is available to authorized users.

## Background

Advanced imaging technologies provide the biologist with considerable insight into the micro-sized embryo, and extend the possibility to conduct research at single-cell level. However, manually analyzing countless cell images is tedious and time-consuming. Automatic image processing becomes essential for exploiting spatiotemporal cellular features [1]. Computer-aided analysis frees biologists from manual work so that they can focus on experiments. Considerable researches on nuclei stack images promote the formulation of biological theories related to nuclear shape and location [2–4]. Membrane, as the physical boundary of the cell, plays a vital role in cell-to-cell communication and development [5–8]. Segmenting clustered cells in 3D, as an important step of image processing, is challenging due to the high-density of cells in the embryo. Although Shan et al. showed remarkable results in 2D cell-shape segmentation [9], the morphology and motion of cell in 3D environments are different from its expression in a single layer 2D image [10–12]. Asan et al. tried to partially stain cells in the embryo, and used cell contours to build a 3D shape model manually [13]. This puts a heavy burden on researchers to annotate a large number of images. Padmini et al. adopted mathematical models and numerical simulations to decode information in cell morphological features [14]. Malte et al. also experimentally demonstrated the dependence between membrane shape and cell communication [15].

*C. elegans* conserves many genes that play significant role in the cell development of advanced animals [16]. More importantly, a *C. elegans* embryo develops via an essentially invariant pattern of divisions, termed as fate determination [17, 18]. The cell division information provides a road map that includes the ancestry and future of each cell at every time point of the development [19]. Therefore, *C. elegans* is used extensively as a model organism to study biological phenomena, such as the genes that influence cell fate decision. It is also important to consider cell shapes during cell division in addition to the timing of the division. Some existing algorithms perform cell morphological segmentation and provide cell shape information, but they are often error-prone on the focal plane, and are exposed to segmentation leakage when the membrane signal is missing. In RACE [20], layer-by-layer results were fused into a 3D cell shape, making RACE a high-throughput cell-shape extractor. However, RACE would segment the membrane surface into one cell instead of interface when the membrane is parallel to the focal plane. This led to the confusing boundaries of two cells in 3D segmentation results. By adding multiple embryos with weak signal, Azuma et al. prevented segmentation leaking into the background in BCOMS [21]. However, the leakage still existed in channel-connected regions caused by the cavity of incomplete membrane surface. Small cavity might lead to totally undistinguishable segmentations.

This paper develops a method for 3D Membrane-based Morphological Segmentation (3DMMS) to extract cell-level embryonic shapes. Novel methods are used to guarantee the precision and robustness of 3DMMS in segmenting a wide range of membrane images. First, intensity degeneration along the slice depth is adjusted statistically through normalization. Hessian matrix transformation is used to enhance the membrane surface signal. Then, a region filter is adopted to remove noisy regions by calculating the location relationship between different components. Subsequently, surface regression is utilized to recover missing surfaces. For the sake of computational efficiency, a membrane-centered segmentation is implemented. Finally, time-lapse fluorescent embryos are segmented at the single-cell level. Combined with the nucleus lineage, 3DMMS can further perform name-based retrieval of cell shape features. Source code is publicly available at [22].

In this paper, “Methods” section presents critical steps in 3DMMS, including pre-processing, membrane-centered watershed segmentation and division correction. “Results” section provides experiment results and a comparison with different algorithms. “Discussion” section explains the advantages and limitations of 3DMMS and points out other possible applications. “Conclusion” section summarizes our contributions and describes our future work.

## Results

Segmentation results from 3DMMS were quantitatively evaluated and compared with two state-of-the-art methods, RACE and BCOMS. To elaborate the performance of 3DMMS, time points with a large number of cells are preferred. However, membrane signal becomes blurry as the number of cells increases, especially for slices at the top of the stack. This prevents experts annotating high-density cells confidently. To enhance the reliability and feasibility of manual annotation, semi-manual segmentation was applied. Six membrane stacks corresponding to time points *t* = 24, 34, 44, 54, 64, 74 were selected. When annotated by experts, all membrane stacks were overlaid with pre-segmentations, which came from nuclei seeded watershed algorithm. After one expert finished the annotation in ITK-SNAP [23], two other experts checked the results individually. All annotations are available at the source code repository.

### Comparison with RACE and BCOMS

To obtain the results from RACE and BCOMS, all images were resampled and resized into 205×285×134. In RACE, parameters, such as *Max 2D Segment area* and *Min 3D Cell Volume*, were tuned for optimal performance. For BCOMS, three consecutive stacks were concatenated into one stack because BCOMS required summing 4D image to generate a single 3D stack for embryonic region segmentation. Only results at the middle time points were used for comparison. For example, we concatenated stacks at *t* = 23, 24, 25 into one stack with size 205×285×402. Slices from 135 to 268 were extracted as the segmentation results of the stack at *t*=24. The reader is recommended to read more details about parameter settings [see “Additional file 1”].

**Fig. 1:**
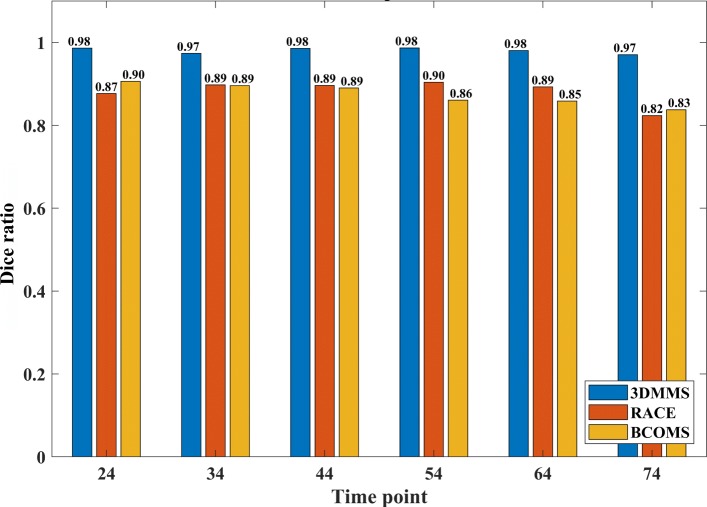
Dice ratio of 3DMMS, RACE, and BCOMS

Dice ratio is universally used in measuring the overlap between the segmentation results *I*_seg_ and ground truth *I*_truth_. In this paper,

1 $$ p=\frac{2\sum\limits_{i=1}^{n}|I_{\text{truth}}^{i}\cap I_{\text{seg}}^{i}|}{\sum\limits_{i=1}^{n}|I_{\text{truth}}^{i}|+|I_{\text{seg}}^{i}|}  $$

is adopted to evaluate the segmentation with multiple cell labels, where *n* is the number of cells in *I*_truth_. Evaluation results are show in Fig. 1. 3DMMS achieves better segmentation precision and robustness over different time points than other methods.

**Fig. 2:**
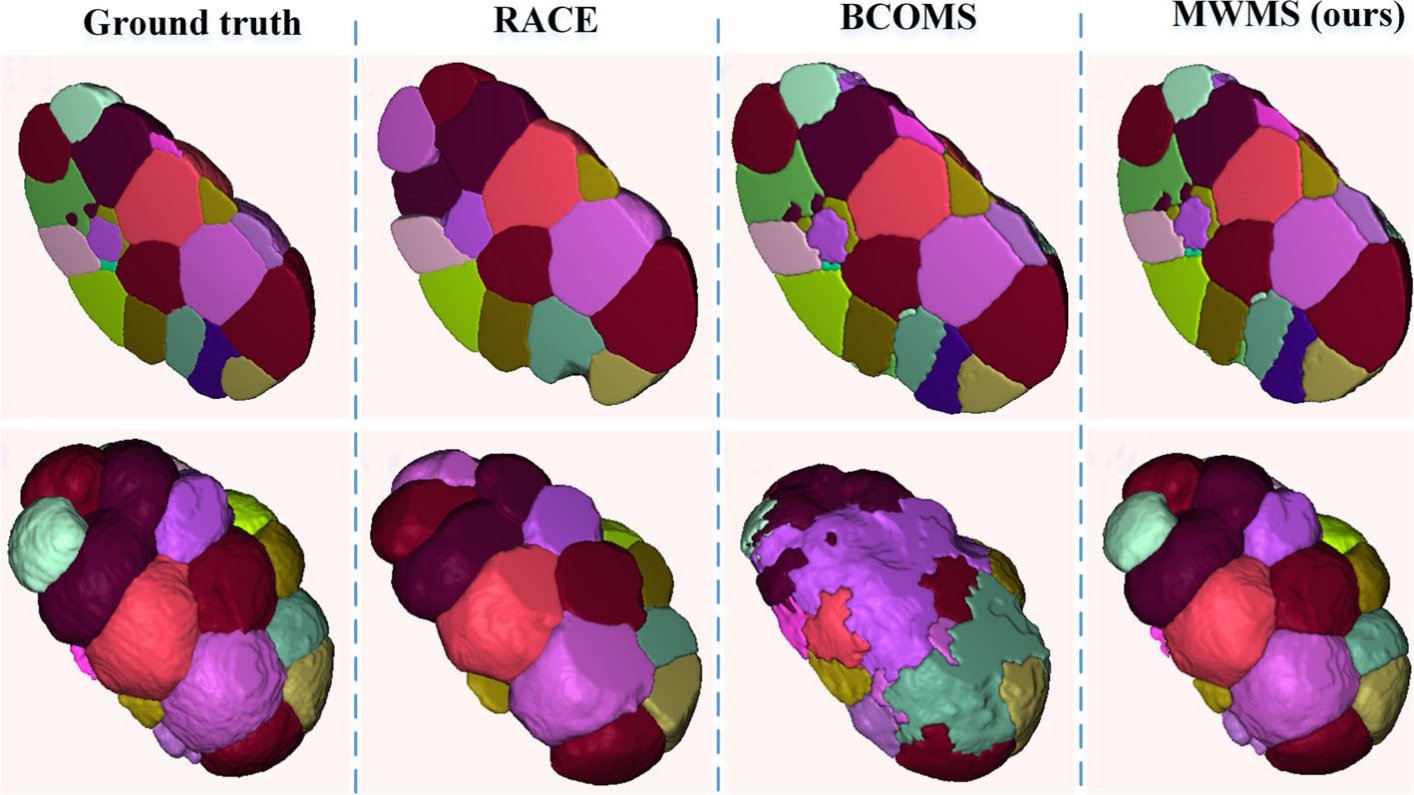
Results comparison. All images come from the same embryo segmentation results. Each column corresponds to the results from the method shown above. Images in the second row are shown in different orientation to images in the first row

**Fig. 3:**
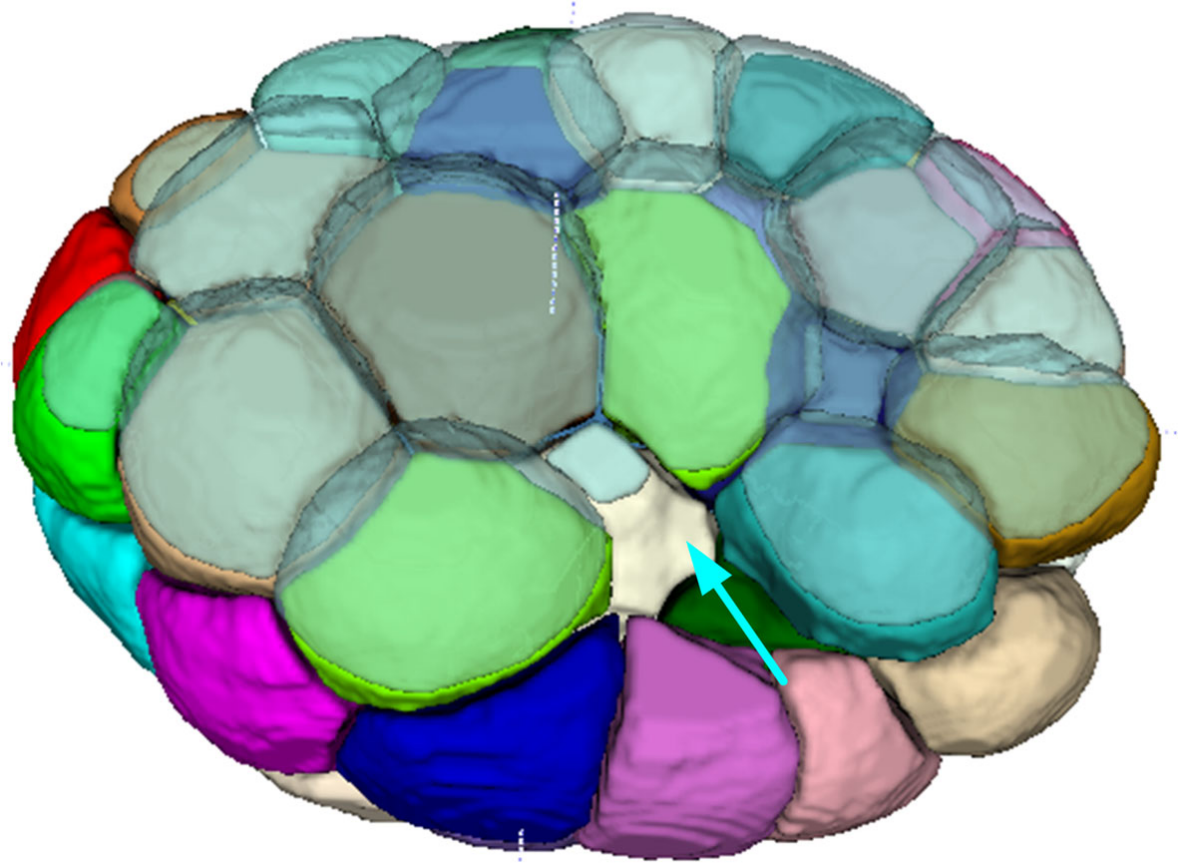
Large gap (cyan arrow) between embryonic surface and eggshell

A deeper insight into the difference among 3DMMS, RACE and BCOMS is illustrated in Fig. 2. RACE provides segmentation with clear and smooth boundaries among neighboring cells. It reconstructs 3D segmentations by fusing results slice-by-slice, making it difficult to distinguish boundaries parallel to the focal plane. In Fig. 2f, cells are sliced off at the top and bottom area. Slice-by-slice segmentation is error-prone in keeping boundary details in 3D because inter-slice information is lost when segmenting a 3D object in 2D. The fusion stage in RACE uniforms labels of fragments, but hardly revises segmentation boundaries. In BCOMS, fewer parameter settings are involved owning to the biological constrains. Moreover, the embryonic eggshell is extracted first to prevent segmented area leaking into the background. This strategy relies on an assumption that the embryonic surface attaches to the eggshell closely. However, the embryonic is not always closely attached to the eggshell, as the manual annotation at *t*=54 in Fig. 3. Constrained by a static eggshell boundary, a cell regions may flow into the gaps between the eggshell and the embryonic surface if a cavity occurs on the embryo surface. 3DMMS shows advantage in both cases, preserving 3D details and diminishing the leakage.

### Segmentation of cells on the boundary

**Fig. 4:**
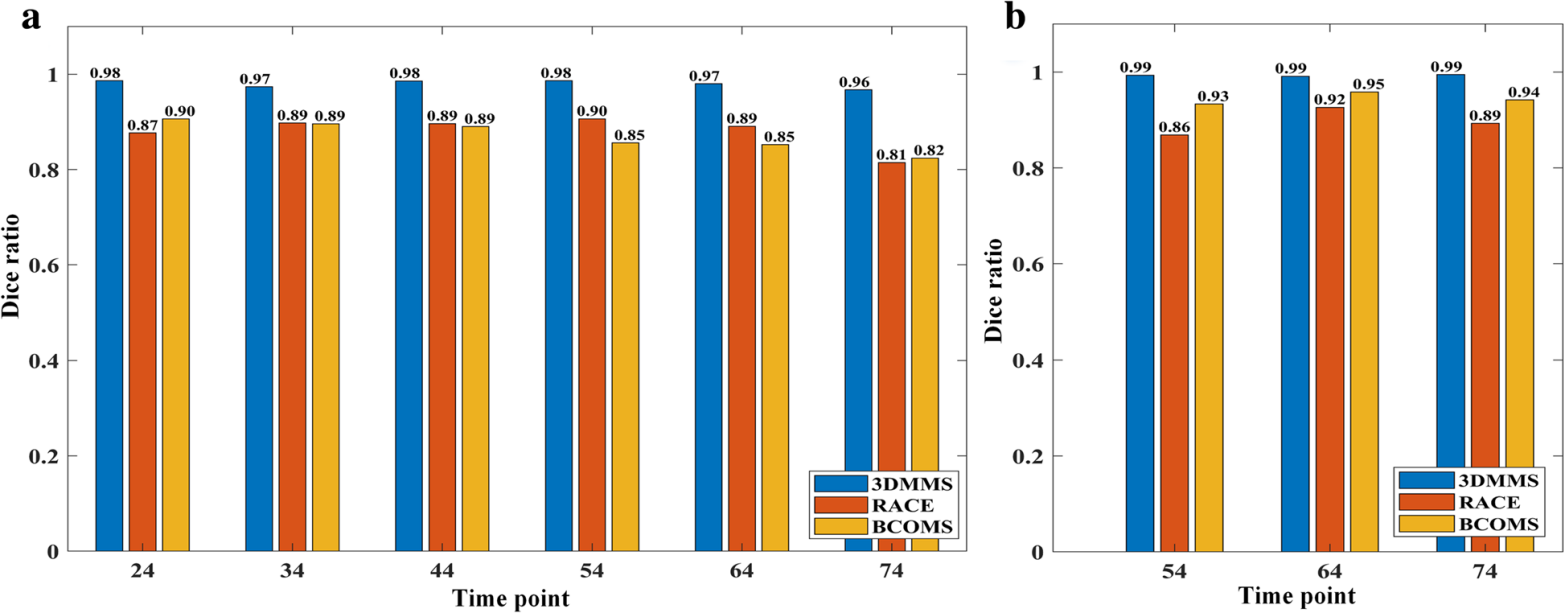
Segmentation precision of cells in the embryo. This figure shows the Dice ratio of segmentation results of cells (**a**) inside and (**b**) at the boundary of the embryo, respectively. All cells contact the background at *t*=24,34,44, so they are not showed in (**b**)

**Fig. 5:**
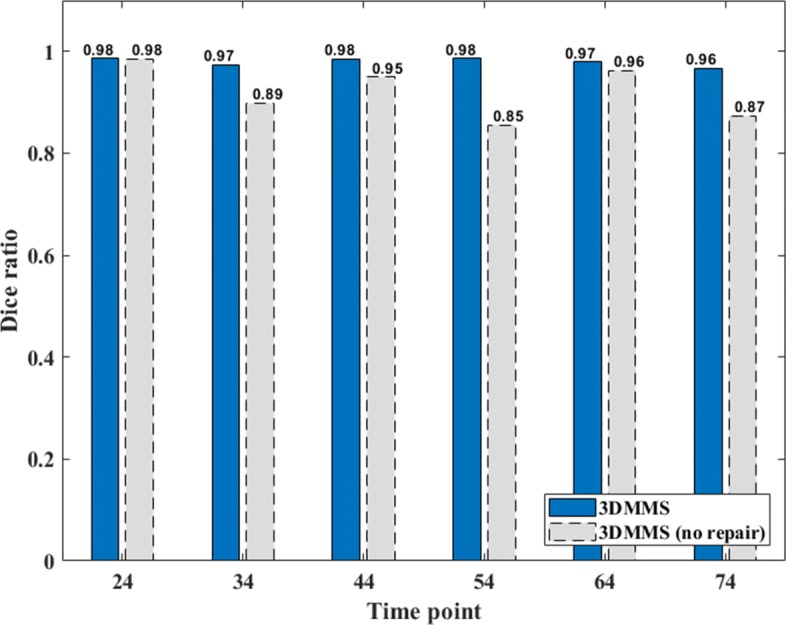
Comparison between 3DMMS with and without cavity repair

During cell imaging, an embryo is stained with a fluorophore and then it is illuminated though a high-energy laser. The membrane signal intensity is determined by the number of photons available to each voxel. The image quality is strongly limited by photo-bleaching, fluorophore concentrations, and small exposure time for acquiring stacks. A membrane image inevitably suffers from the lost information, especially for cells at the boundary of the embryo. Incomplete embryonic surface is a major factor influencing the overall precision. To check the accuracy of the segmentation on the boundary cells, we calculated the Dice ratio corresponding to cells inside and at the boundary of the embryo, respectively, as shown in Fig. 4. Comparing Figs. 4a and b, we find that three methods produce a higher Dice ratio inside the embryo, particularly for BCOMS. This observation meets our expectations because inside the embryo, the image has a higher signal-to-noise ratio. The primary error of BCOMS originates from the leakage around the embryonic surface. In 3DMMS, embryonic surface is well repaired in the surface regression procedure, effectively preventing cell region flooding into the background. To emphasize the necessity of repairing cavity in Fig. 4a, the Dice ratio of the results from 3DMMS without cavity repair is also shown in Fig. 5.

## Discussion

In “Results” section, 3DMMS is compared with two state-of-the-art methods. 3DMMS provides better segmentation results of the whole embryo. Note that our contributions focus on processing membrane stack images and producing 3D embryo structure. In order to elaborate the benefits of 3DMMS fully, nucleus lineage information is utilized from *AceTree* [24]. After integrating cell shapes into the lineage, researchers can not only obtain cell morphological features, such as volume, surface area and neighboring cells, but also make a longitudinal comparison of cellular shapes. To our best knowledge, 3DMMS is the first software that can achieve the cell-name-based retrieve for shape features, such as volumes and interface between neighboring cells. This dramatically expends our study from the nucleus to the whole cell. In this section, we will discuss other potential applications of 3DMMS.

### Applications to the study of internal features

**Fig. 6:**
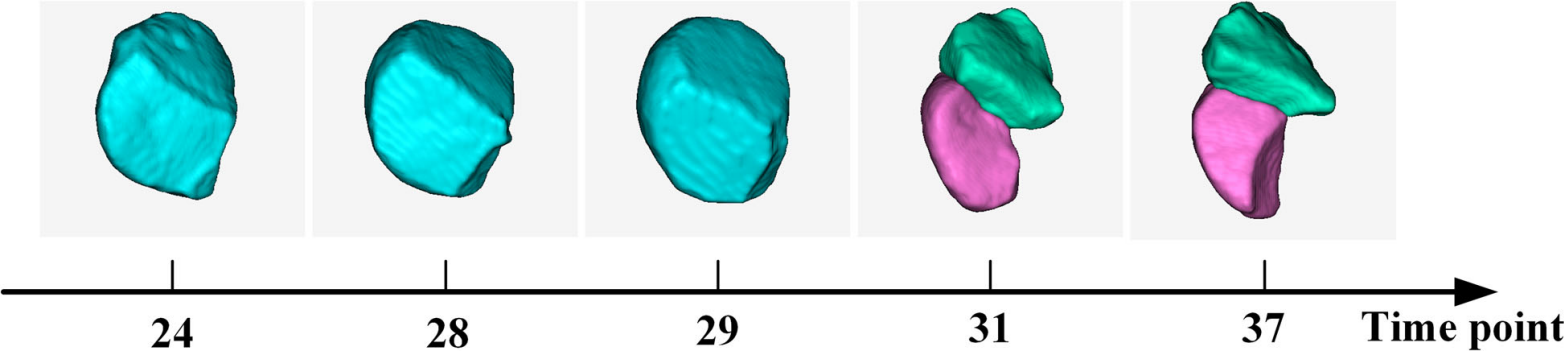
Morphological deformation of cell “ABala” during division

Recent studies indicate that gene expression and protein synthesis are influenced by the nuclear shape [25]. In fact, 3DMMS can provide a way to study whether biological expression modulates cell shapes. Previous algorithms are designed for either individual cell image or time-lapse nucleus image. They neglect the shape deformation of a cell with time. Although *AceTree* provides cell trajectory, it is limited to the nuclei without any cell shape information. Segmentation in 3D is essential for tracking the whole dynamic cell across multiple slices. With the cell shape lineage, we can track time series of cellular shape deformation. One cell division process is demonstrated in Fig. 6 as an example. Thus, our method is useful for the study of temporal morphological deformations of cells.

### Applications to the study of external features

**Fig. 7:**
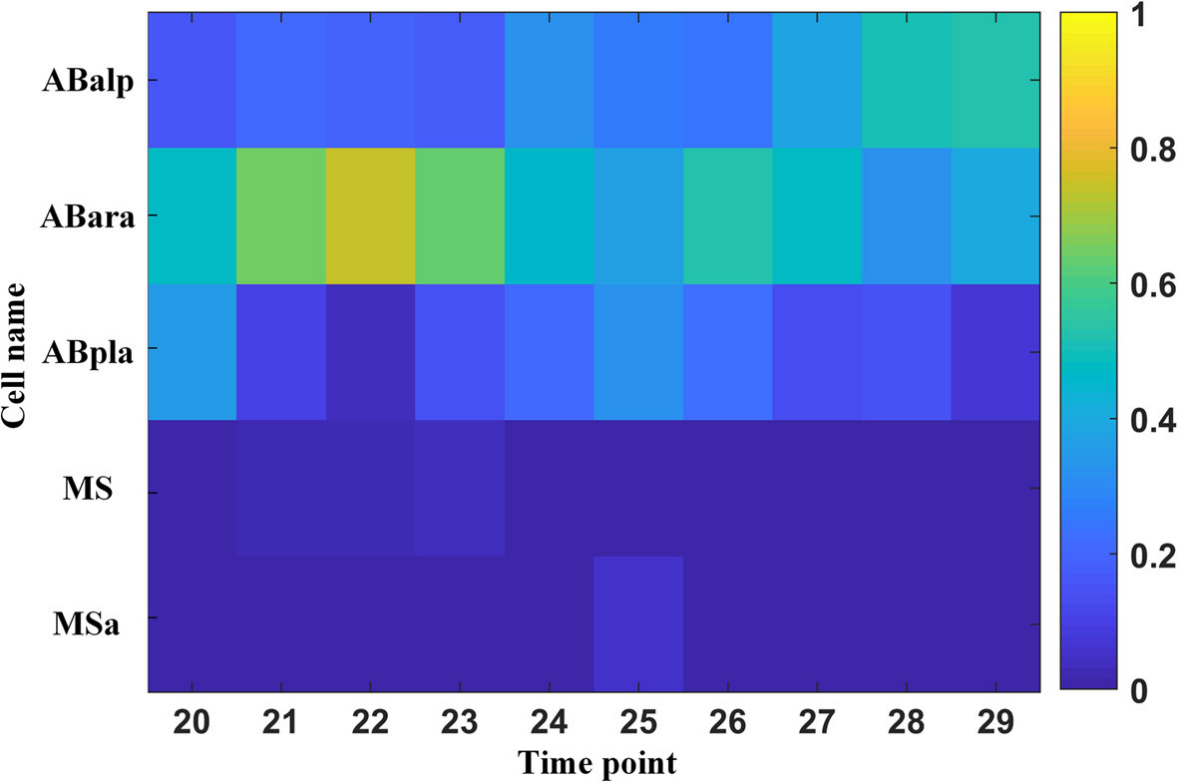
Interface matrix between cell “ABala” and its neighboring cells. The sum of each column equals to 1. Every element represents the ratio of the interface between one cell and “ABala”, to the overall interface

Ratajczak et al. reported that information can be transferred through cell membrane, further affecting the cell’s development [26]. Various works have qualitatively analyzed the communication between cells, but few of them were involved in measuring the interface of two cells. Statistical analysis is also needed to enhance the reliability of shape deformation. It leads to a demand for the 3D shape information in 3DMMS. With the region of each cell clearly identified, we can easily infer cell’s contextual information, such as neighboring cells. Example in Fig. 7 presents the interface ratio of cell “ABala” to its neighboring cells.

### Applications to other types of images

This paper utilizes *C. elegans* to explain the implementation of 3DMMS. However, methods in 3DMMS are not confined to the segmentation of C. elegans embryos. Our algorithm provides a systematic procedure for cell segmentation. No assumptions dependent on C. elegans are made in the entire process. With algorithms, such as TGMM [27], MaMuT [28], which can produce the cell lineage of other similar embryos, 3DMMS can be also used to exploit other kinds of cell’s morphological features.

### Weakness of the 3DMMS

**Fig. 8:**
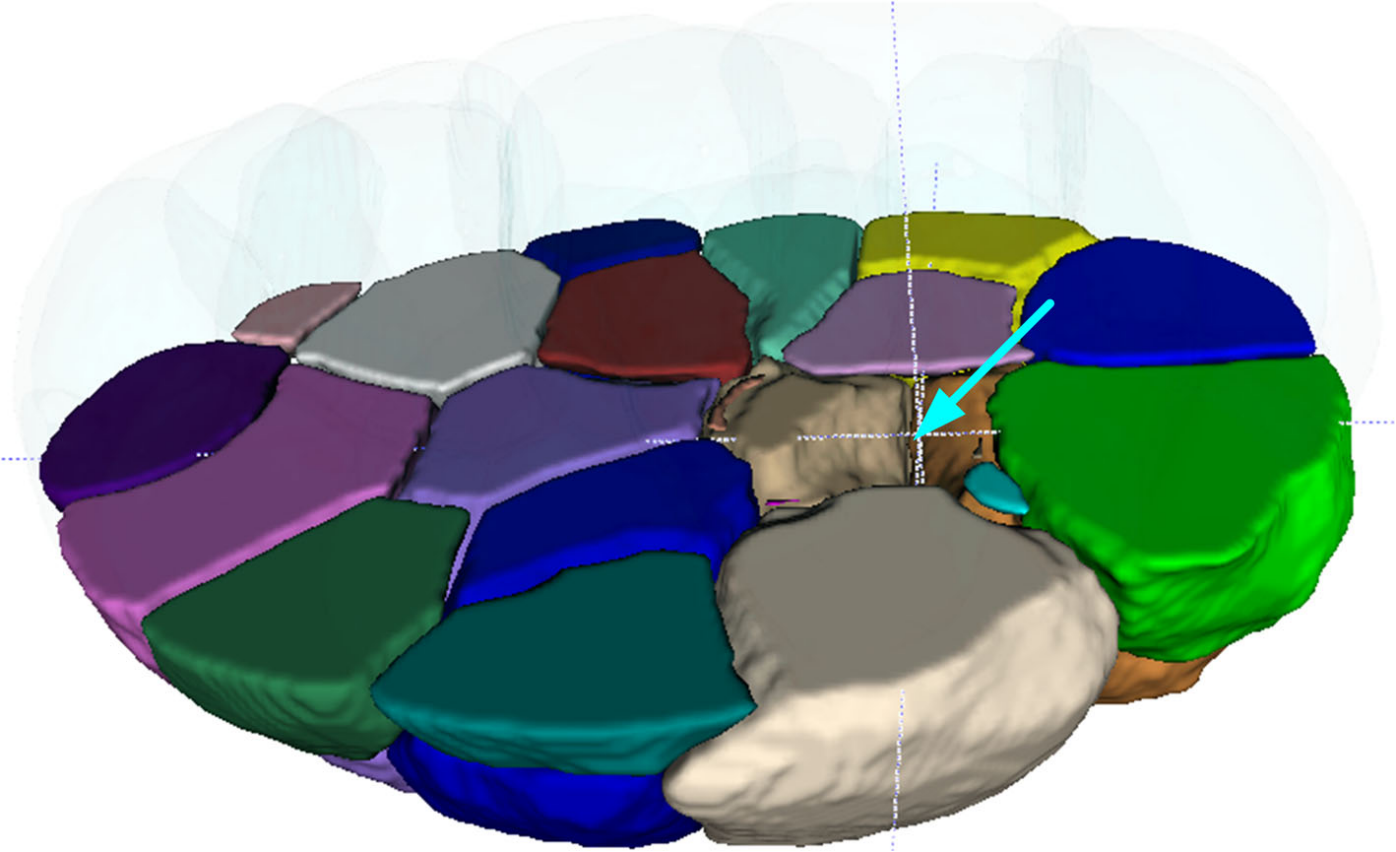
Gap (cyan arrow) between cells inside the embryo

Based on the watershed algorithm, 3DMMS builds boundary lines if and only if two basins contact with each other. Therefore, 3DMMS might fail to detect gaps inside the embryo. In our experiments, most of cells were closely attached to its neighbors. However, some cases did appear where small gap arose among neighboring cells, as shown in Fig. 8. We will conduct much more experiments and study different configurations of various gaps to improve the performance of 3DMMS in the future.

## Conclusion

This paper reports an effective method based on 3DMMS to analyze embryonic morphological features at the single-cell level. 3DMMS is robust and can adapt to images at different time points. Based on this method, it is feasible to analyze cell shape longitudinally and transversally. Our future work will include designing specific geometric model, such as the formulation proposed by Kalinin *et al* [29]. Then, we will carry out statistical analysis on a large dataset of *C. elegans* embryos. We envision that 3DMMS could help biologists investigate morphological features related to biological regulations.

## Methods

**Fig. 9:**
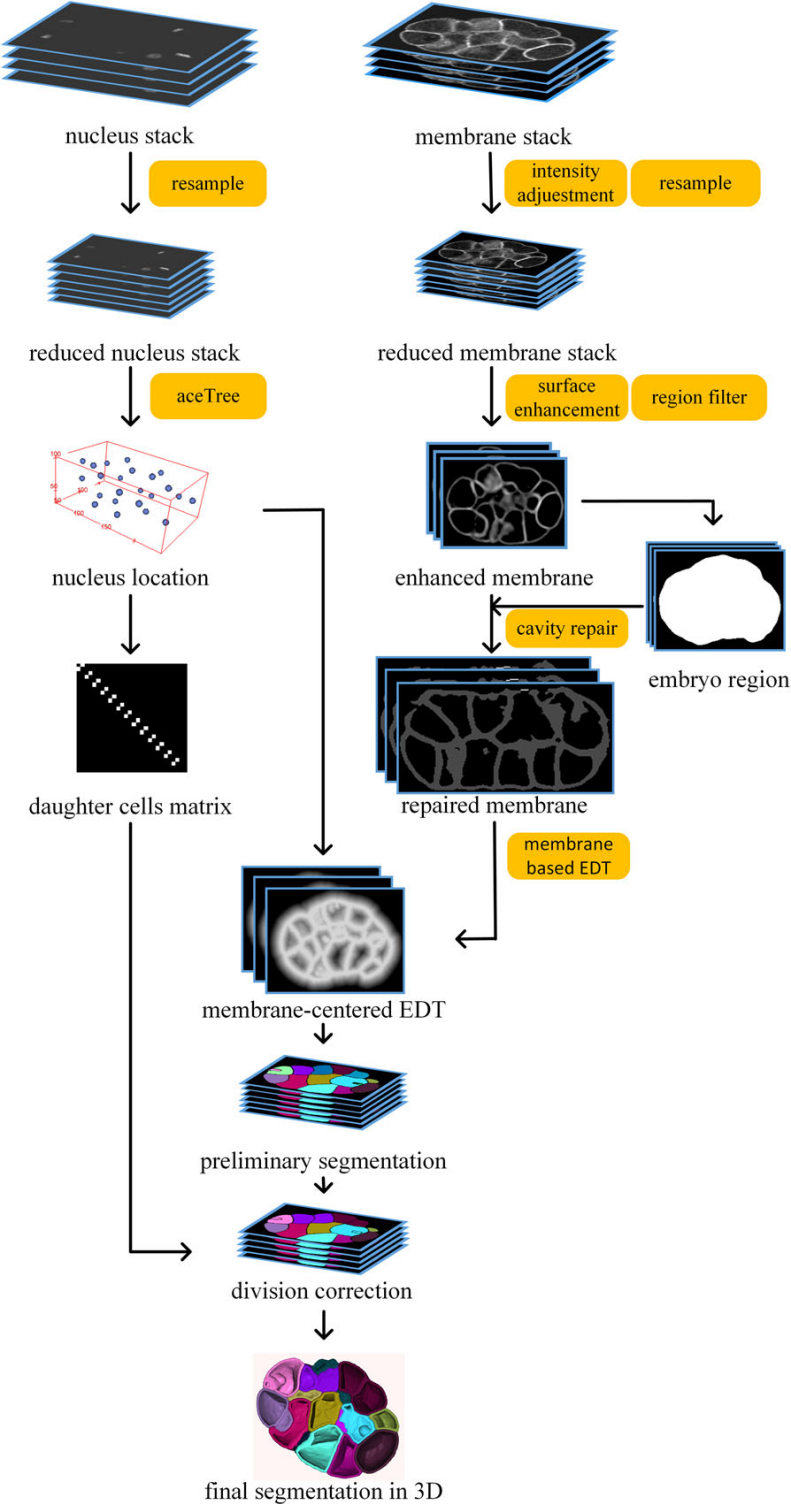
Flowchart of our methodology

Optical appearance of cell membrane is variable due to different size, number, and position of fluorescent signals on the focal plane. In our method, a membrane image is preprocessed with multiple steps. A fluorescent microscope produces membrane stack (red) and nucleus stack (blue) simultaneously. While nucleus channel is used to generate (nucleus-level) seeds matrix by existing methods, we obtain the cellular shapes by leveraging the membrane channel. The framework of 3DMMS can be divided into three parts, membrane image preprocessing, membrane-centered segmentation and division correction, as illustrated in Fig. 9.

### Data

*C. elegans* was first stained with dual labelling in cell nucleus and membrane. All the animals were maintained on NGM plates seeded with OP50 at room temperature unless stated otherwise. Membrane marker and lineaging marker were rendered homozygous for automated lineaging. To improve the overall resolution, 4D imaging stacks were sequentially collected on both green and red fluorescent protein (mCherry) channels at a 1.5-min interval for 240 time points, using a Leica SP8 confocal microscope with a 70-slice resonance scanner. All images were acquired with resolutions of 512×712×70 stack (with voxel size 0.09×0.09×0.43*μ*m). All the images were deconvoluted and resized into 205×285×70 before analysis.

### Membrane image preprocessing

#### Statistical intensity normalization

**Fig. 10:**
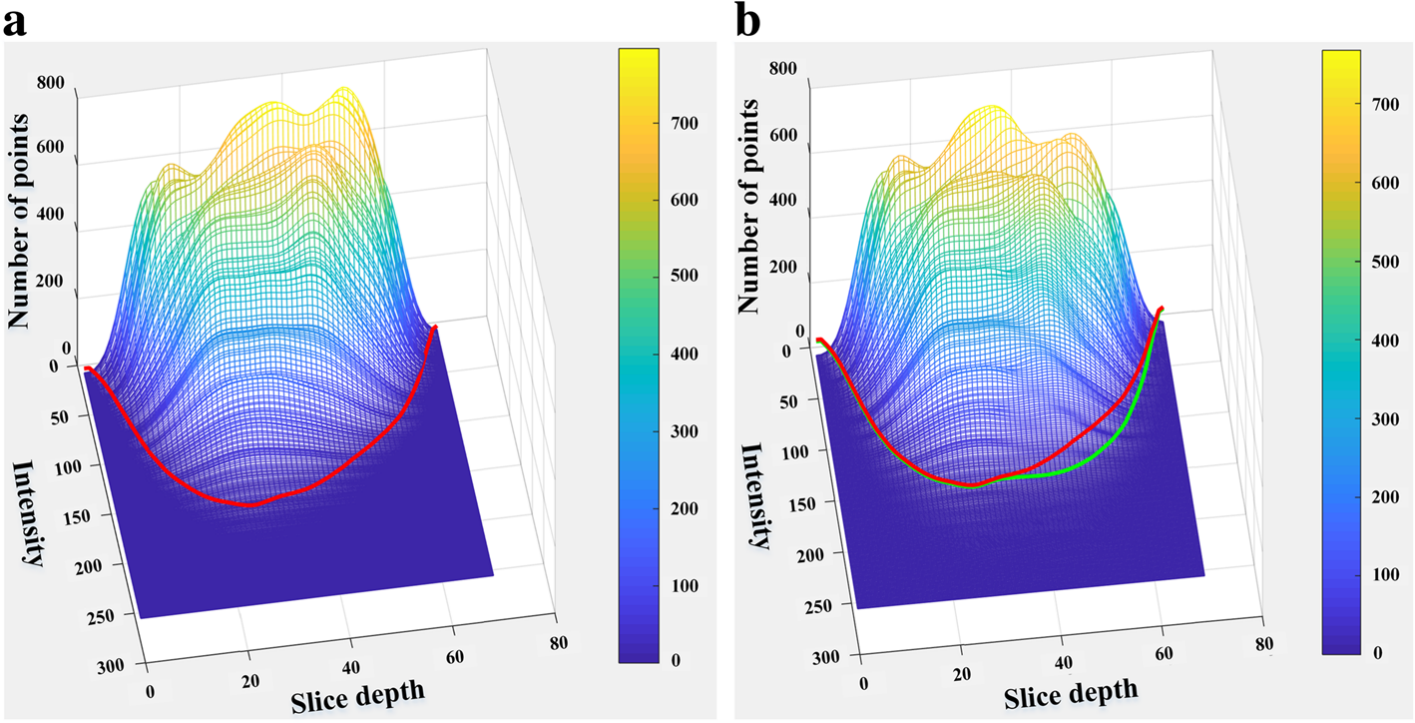
Slice intensity distribution matrix. **a** Intensity matrix before adjustment with red threshold line; **b** Intensity matrix after adjustment with green threshold line. Red line in (**a**) is also plotted for comparison. Both red and green lines correspond to the same threshold on “Number of points”

Fluorescent images are often corrupted by noise, such as Poisson distributed incoming photos. Besides, signal intensity decreases along the *z*-axis because of the attenuation of laser energy. To achieve parameter generalization through the whole stack, Gaussian smoothed membrane image was adjusted by statistical intensity normalization, which balanced the intensity distribution of symmetrical slices in each stack. First, pixel intensity histogram of each slice was embedded into an intensity distribution matrix as a row. Background pixels were ignored for computational stability. An example of Gaussian smoothed intensity distribution matrix is shown in Fig. 10a. A threshold of the pixel number was applied, thus a threshold line (red in Fig. 10a) was formed across all slices. Slices at the deeper half of the stack were multiplied by the ratio of this slice’s intensity on the red line to that of its symmetrical slice. The stack intensity distribution after the adjustment is shown in Fig. 10b.

Additionally, the membrane stack was resampled to 205×285×134 with linear interpolation on the *z*-axis.

#### Hessian matrix enhancement

Cell surfaces are composed of plane components. Membrane signals can be enhanced by selecting all pixels that belong to a plane structure. We took the associate quadratic form to exploit intensity changes surrounding a pixel, and further determined its structure components. By diagonalizing the quadratic form, the Hessian descriptor is defined as

2 $$ {{} \begin{aligned} H\,=\,\left[ \begin{array}{ccc} \frac{\partial^{2}{I^{\mathrm{m}}}}{\partial{x^{2}}} &\frac{\partial^{2}{I^{\mathrm{m}}}}{\partial{xy}} &\frac{\partial^{2}{I^{\mathrm{m}}}}{\partial{xz}}\\ \frac{\partial^{2}{I^{\mathrm{m}}}}{\partial{yx}} &\frac{\partial^{2}{I^{\mathrm{m}}}}{\partial{y^{2}}} &\frac{\partial^{2}{I^{\mathrm{m}}}}{\partial{yz}}\\ \frac{\partial^{2}{I^{\mathrm{m}}}}{\partial{zx}} &\frac{\partial^{2}{I^{\mathrm{m}}}}{\partial{zy}} &\frac{\partial^{2}{I^{\mathrm{m}}}}{\partial{z^{2}}} \end{array} \right]\,=\,\left[\begin{array}{ccc} \vec{e_{1}} &\vec{e_{2}} &\vec{e_{3}} \end{array} \right]\left[\begin{array}{ccc} \lambda_{1} &0 &0\\ 0 &\lambda_{2} &0\\ 0 &0 &\lambda_{3} \end{array} \right]\!\left[\begin{array}{c} \vec{e_{1}}\\\vec{e_{2}}\\\vec{e_{3}} \end{array} \right] \end{aligned}}  $$

where *λ*_1_,*λ*_2_,*λ*_3_ are eigenvalues with |*λ*_1_|<|*λ*_2_|<|*λ*_3_|, and $\vec {e_{1}}, \vec {e_{2}}, \vec {e_{3}}$ are the corresponding eigenvectors. Pixels could be allocated to three structures regarding the eigenvalues: (1) when |*λ*_1_|,|*λ*_2_|<1 and |*λ*_3_|≥1, the pixel locates on a plane; (2) when |*λ*_1_|<1 and |*λ*_2_|,|*λ*_3_|≥1, the point locates on a stick; and (3) when |*λ*_1_|,|*λ*_2_|,|*λ*_3_|≥1, the point locates in a ball. So membrane surface signal can be enhanced with

3 $$ I^{\text{en}}(x,y,z)=\frac{|\lambda_{3}(x,y,z)|}{\max\left(|\lambda_{3}(x,y,z)|x,y,z\in{\text{stack voxels}}\right)}  $$

where *I*^en^ is the stack image after enhancement.

#### Region filter

Preliminary experiment shows membrane based EDT (in “Membrane-centered segmentation” section) is highly dependent on the quality of binary membrane image. The region filter is designed to screen noise regions in *I*^en^. After suppressing noise and enhancing membrane signal, we choose a threshold to convert *I*^en^ into binary image *I*^bn^. It is composed of disconnected regions, denoted as *Φ*={*ϕ*_*i*_}, some of which are noise spots. The largest connected region *ϕ*_*i*_ belongs to valid cell surface signal *χ*, but other regions need to be screened. Keeping noise spots would introduce erroneous cell boundaries, whereas missing valid signal results in segmentation leakages.

Herein, principal component analysis (PCA) was employed to analyze the location relationship between *ϕ*_max_ and small regions in {*Φ*∖*ϕ*_max_}. Noise and valid regions had different influence on the Euclidean distance transformation (EDT) of the membrane surface *ϕ*_max_. The flow chart of the region filter is shown in Fig. 11. Cell surface signal was initialized as *χ*={*ϕ*_max_}. Following steps were repeatedly used to update *χ*:

1. Construct zero matrix *L* with the same size as *I*^bn^. Points already in *ϕ*_max_ are set as 1 in *L*. *DL* denotes the EDT results on *L*. Similarly, after another region *ϕ*_*i*_ (green or yellow region in Figs. 11b and d) in {*ϕ*∖*χ*} is combined into *L*, EDT is also used to generate *DL*^′^.

**Fig. 11:**
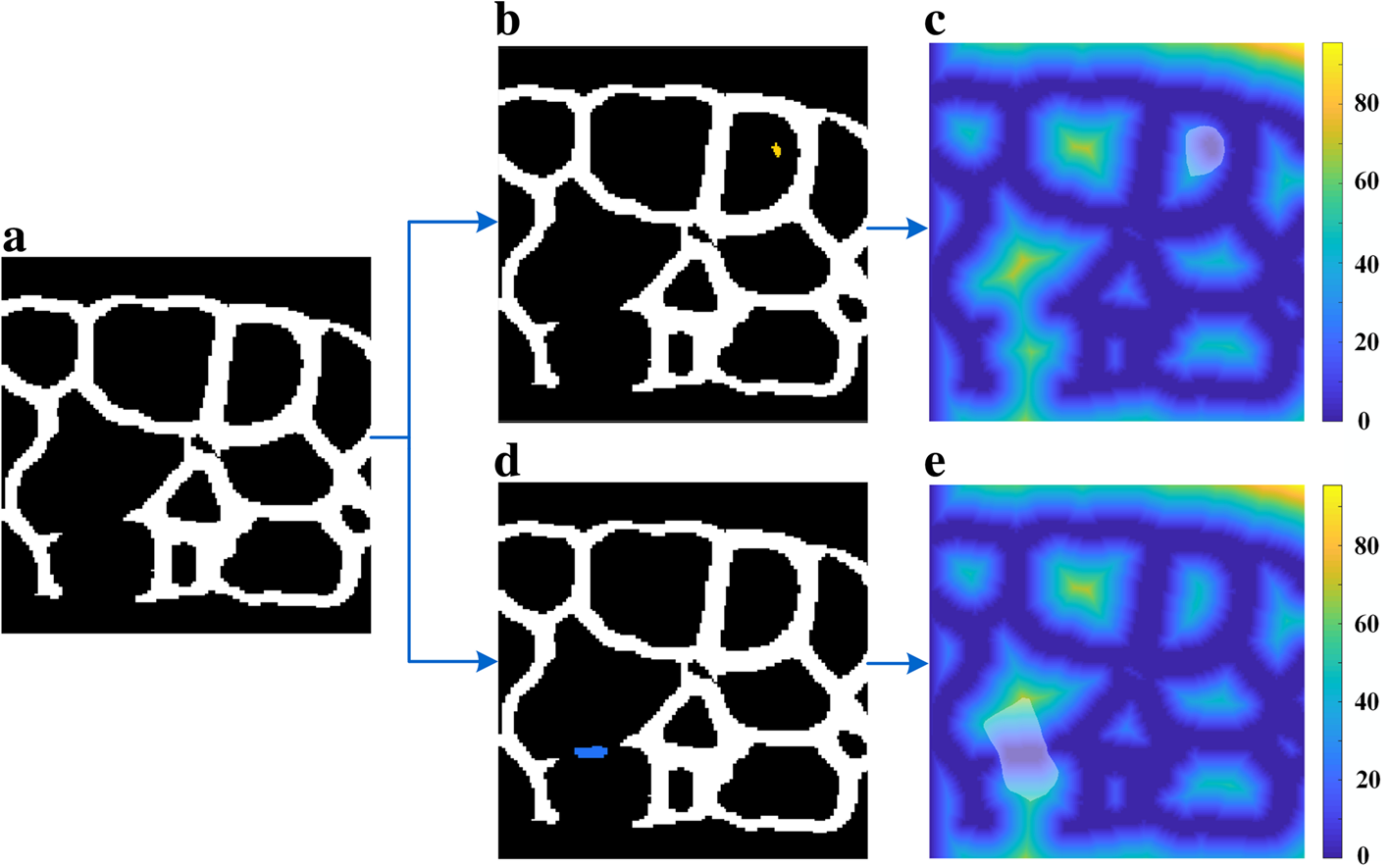
Influence of noise spot and valid membrane region on the EDT of membrane surface. This figure includes steps in region filter. **a** Largest membrane surface *ϕ*_max_; **b** Add noise spot *ϕ*_*i*_ to *ϕ*_max_; **c** EDT of noise and *ϕ*_max_; **d** Add valid membrane *ϕ*_*i*_ to *ϕ*_max_; **e** EDT of membrane and *ϕ*_max_. Path (**a**)-(**b**)-(**c**) shows when a noise spot is added into the largest membrane surface, the influenced region R (transparent white mask in (**c**) and (**e**)) in the EDT tends to be round. Conversely, Path (**a**)-(**d**)-(**e**) indicates if a valid membrane region is added into the membrane surface, the influenced region has notable polarization. Note that noise spot (yellow in (**b**)) and valid membrane region (blue in (**d**)) all exist in binary filtered membrane *I*^bn^, but shown here separately for better demonstration
2. We use
  4 $$ R=\left\{(x,y,z)|DL(x,y,z)\neq DL'(x,y,z)\right\}  $$
  to obtain the influenced EDT region *R* when we add *ϕ*_*i*_ into *L*.
3. Use PCA to analyze the polarization features of *R*. Variance percentage on three directions are *γ*_1_,*γ*_2_,*γ*_3_ and *γ*_1_<*γ*_2_<*γ*_3_. The coefficient for adding *ϕ*_*i*_ into *χ* is measured by $\frac {\gamma _{1}}{\gamma _{1}+\gamma _{2}+\gamma _{3}}$. Our experiments shows that if this coefficient is larger than 0.1, *ϕ*_*i*_ can be regarded as membrane signal and should be grouped into *χ*. Otherwise, *ϕ*_*i*_ will be ignored.

**Fig. 12:**
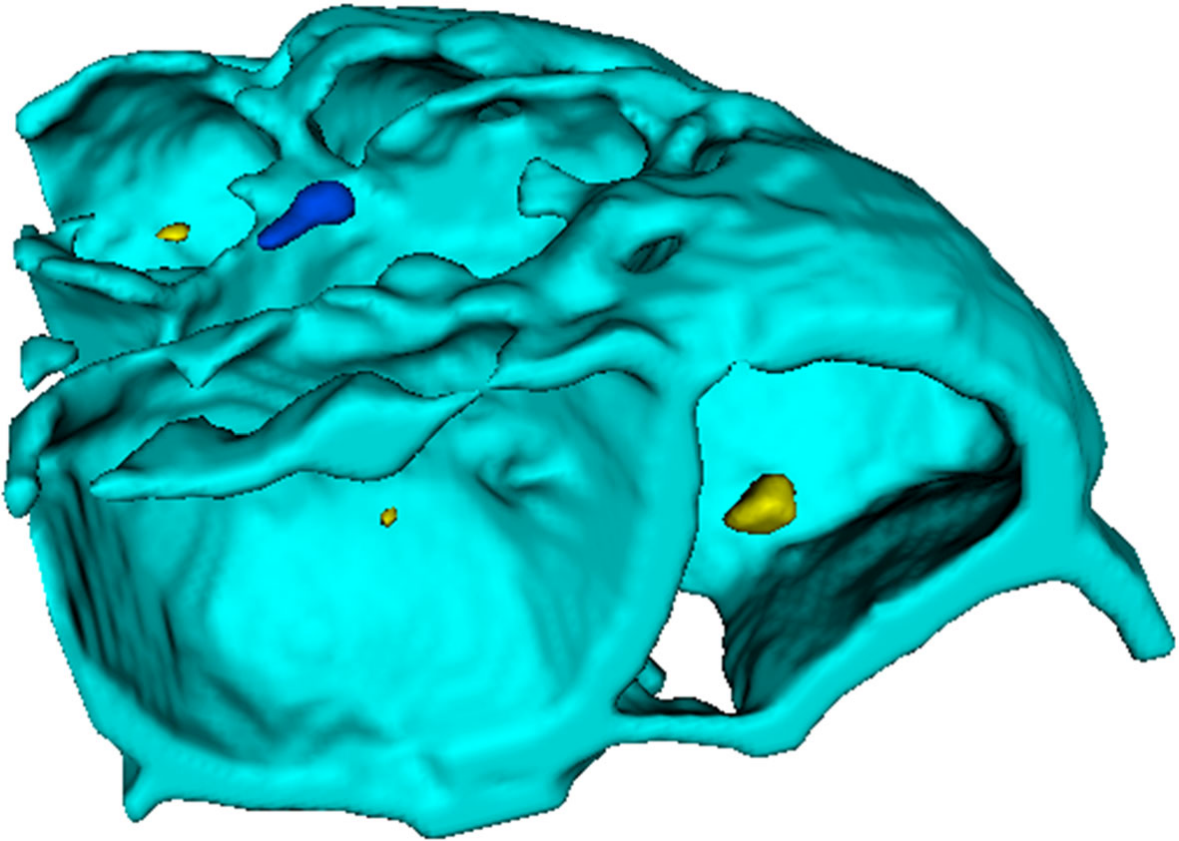
Results obtained using the region filter. Results processed by region filter, where blue and yellow regions represent valid membrane signal and noise spots, respectively

An example result is shown in Fig. 12. Filtered membrane stack *I*^fm^ is a binary image whose points in *χ* is positive.

#### Surface regression

**Fig. 13:**
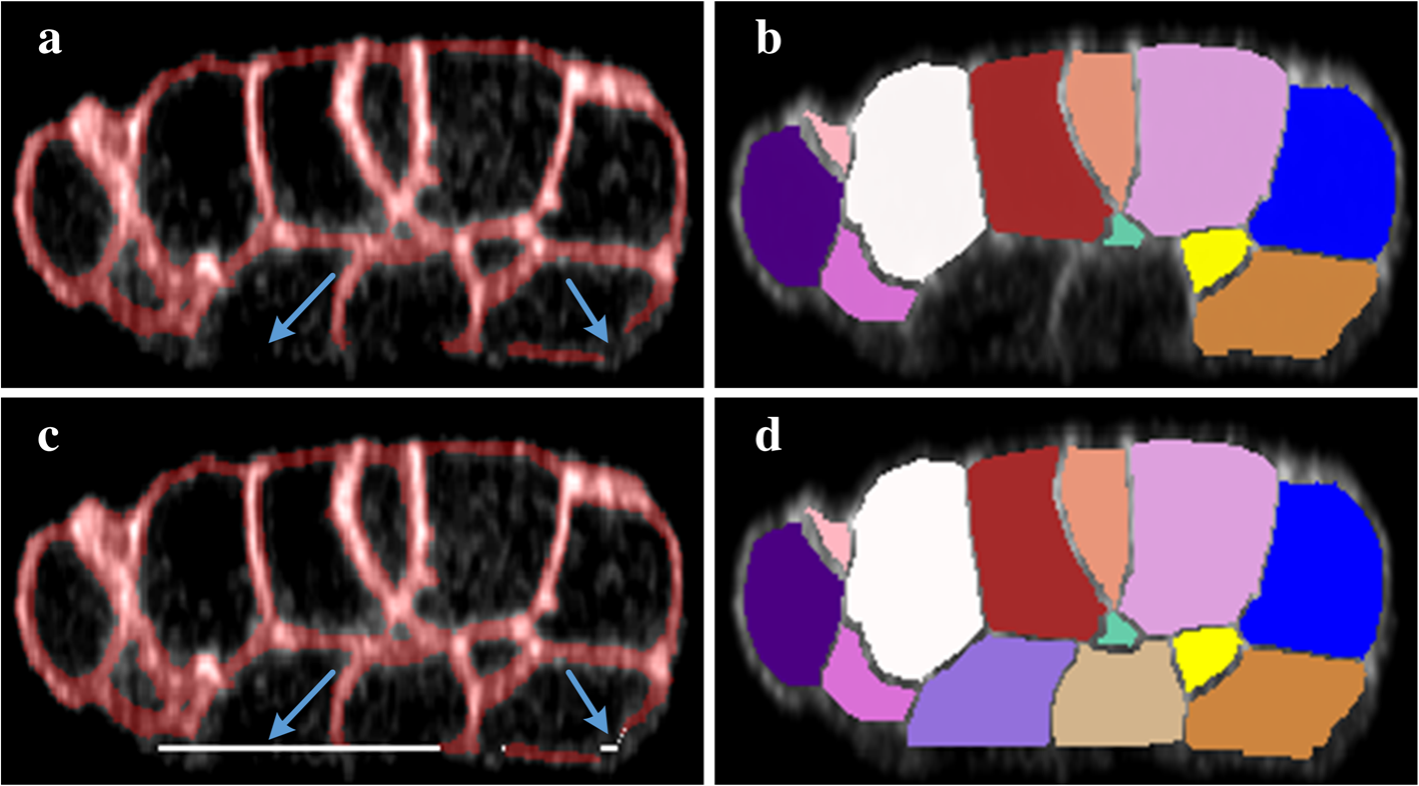
Surface regression on cavity. Binary image (red region in (**a**)) suffers from lost membrane surface. **b** is the segmentation results from (**a**). Two cells are lost because of the background leakage to the embryo. Cavities are repaired with surface regression in (**c**), preventing background flowing into the background

The embryonic surface cannot be imaged completely because of a balance between the phototoxicity and signal intensity. Moreover, the stain concentration is much lower at the boundary where only one layer of the membrane exists. Incomplete surface degrades the performance of 3DMMS because of the leakage between different targets, as shown in Fig. 13b. We use surface regression to recover the boundary surface signal around the missing embryonic surface area, noted as surface cavity. In surface regression, we only modify surfaces in the cavities and this is different from the embryonic region segmentation in BCOMS.

**Fig. 14:**
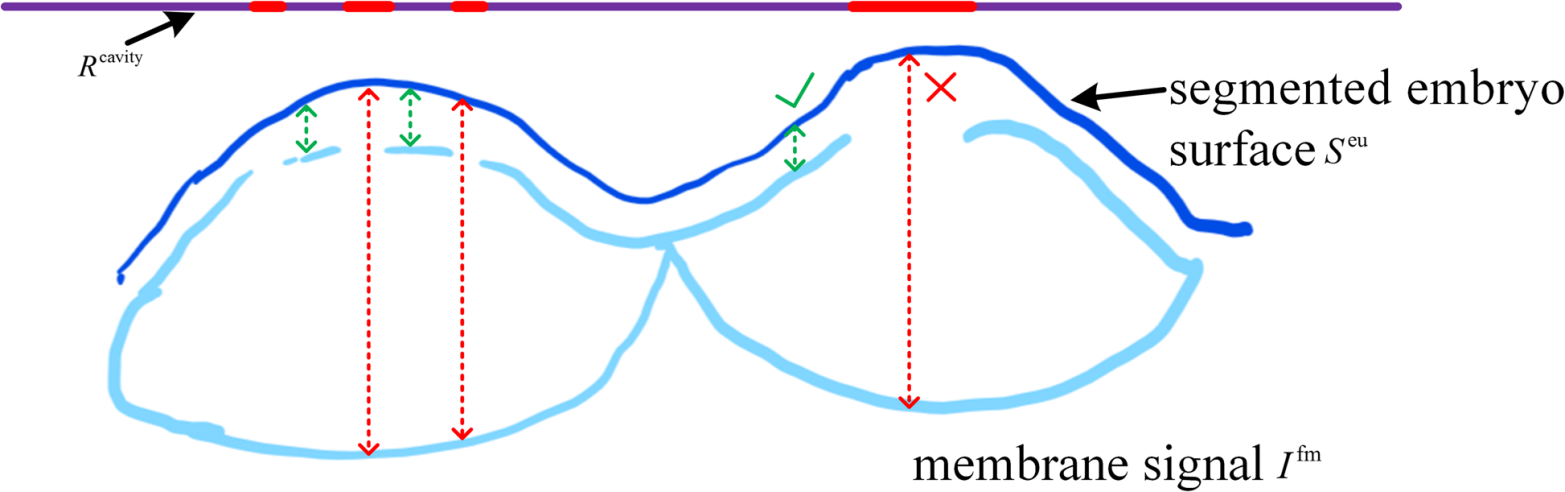
A graphical explanation of surface cavity repair. Dot lines represent the distance between segmented embryo surface *S*^eu^ and membrane signal *I*^fm^. Pixels with large distance are projected to binary mask *R*^cavity^ with positive values

We apply the active surface first to obtain the initial surface of the entire embryo. The smooth factor is tuned to be a large value to prevent segmented surface dropping into the cavity. From Fig. 14, we know that cavity surface can be found according to the vertical distance between the segmented embryo surface and the membrane signal *I*^fm^. We defined a distance matrix as the same size as one slice. For the upper half surface of the segmented embryonic surface *S*^eu^, the distance matrix delineated the vertical distance between *S*^eu^ and membrane signal *I*^fm^. The distance was set to zero when there were no corresponding signals. Distance matrix was smoothed, and further thresholded using Ostu’s method [30], to construct a binary mask *R*^cavity^. Positive masks in *R*^cavity^ indicated the location where the membrane signal should be modified with *S*^eu^. We used

5 $$ I^{\text{fm}}\left(x,y,S^{eu}(x,y)\right)= \begin{cases} 1, &\text{if }R^{\text{cavity}}(x,y)=1\\ 0, &\text{if }R^{\text{cavity}}(x,y)\neq 1 \end{cases}  $$

to repair *I*^fm^. Partial surfaces with positive mask were added into *I*^fm^, shown as gray points in Fig. 13c.

### Membrane-centered segmentation

Watershed segmentation is a fast algorithm to group points with different labels according to specific terrain map based on image intensity. Along the steepest descent, all pixels are classified into different catchment basin regions by tracing points down to the corresponding local minima [31], which are also termed as seeds. After watershed transformation, each region consists of points whose geodesic descent paths terminate at the same seed. The number of seeds controls the number of regions. Redundant seeds result in over-segmentation where one region is split; whereas, absent seeds lead to under-segmentation with two regions combined together. The terrain map plays a dominant role in generating region boundaries. In 3DMMS, a well-defined terrain map, combined with nucleus channel, accommodates the difficulty of lost information and membrane perception.

**Fig. 15:**
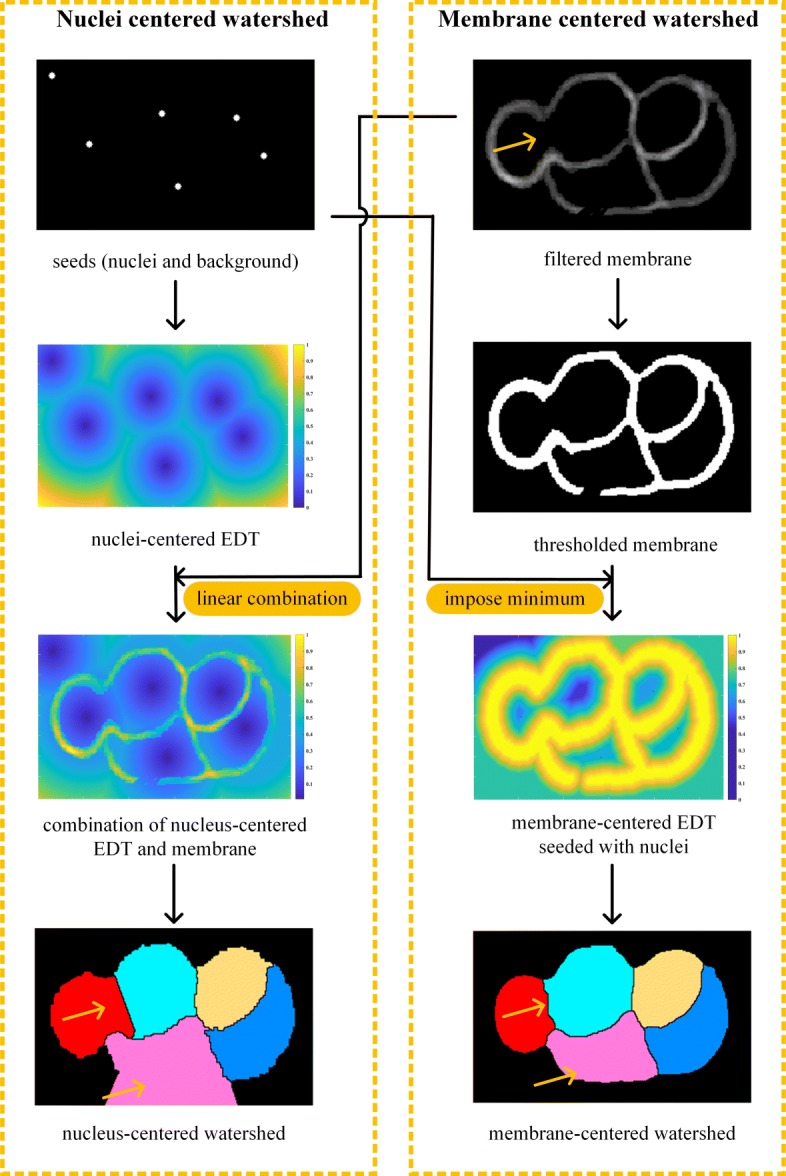
Comparison between nucleus-centered and membrane-centered watershed segmentation

The nucleus image is simultaneously acquired with the membrane image, which can be used as seeds to eliminate merge-or-split mistakes. Generally, the terrain map is the linear combination of membrane intensity in nucleus-centered watershed segmentation [21*,*^32^*–*34]. However, it is difficult to make a tradeoff between two sources of influence on the final region boundary, as shown in Fig. 15 (*combination of EDT and membrane*). To overcome this problem, we combined nucleus and membrane stacks in a different way, noted as membrane-centered watershed. The nucleus stack was processed by *AceTree* to generate the nucleus matrix. The nucleus matrix *I*^n^ was constructed as

6 $$ I^{\mathrm{n}}=l_{i}  $$

where (*x*_*i*_,*y*_*i*_,*z*_*i*_) and *l*_*i*_ were the nucleus location and label in the lineage, respectively. We noted *D*^m^ as the membrane-centered EDT on *I*^fm^. Then *D*^m^ was reversed and normalized by

7 $$ D^{\mathrm{m}}=\frac{\max(D^{\mathrm{m}})-D^{\mathrm{m}}}{\max(D^{\mathrm{m}})}  $$

The nucleus matrix *I*^n^, plus a background minimum, were used as seeds for the watershed segmentation on new terrain map *D*^m^. This map can, to a certain extent, relieve the segmentation leakage by building a ridge at the holes of the binary membrane signal, as demonstrated in Fig. 15 (*membrane-centered EDT*). Channel-connected cells were well separated with each other. It produces reasonable boundaries in both the blurry area and surface cavities.

### Cell division revision

**Fig. 16:**
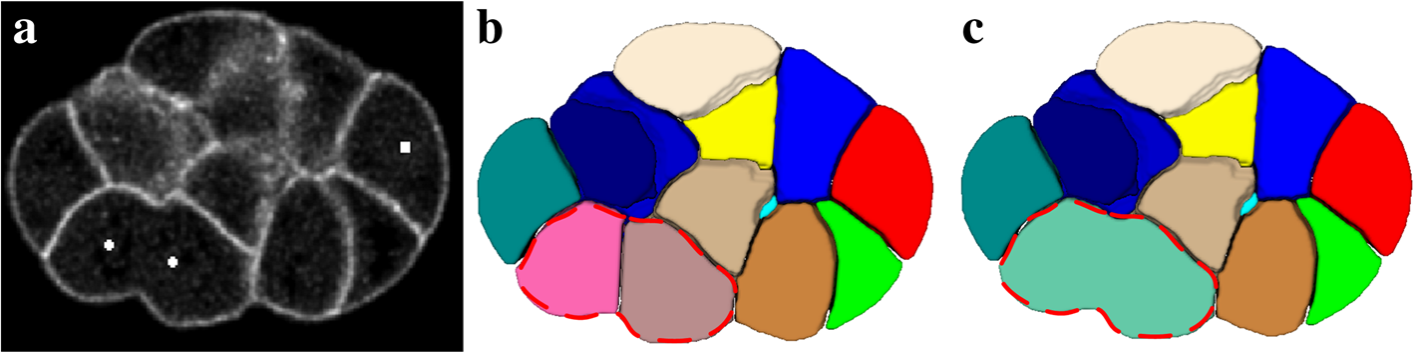
Example in division correction **a** Raw membrane image; **b** Segmentation before correction; **c** Segmentation after correction

Two nuclei in a dividing cell would lead to a split, indicated with red lines in Fig. 16b. We resolved this problem by considering the membrane signal distribution of the interface between two cells. First, we analyzed nucleus lineage information and found out the daughter cells (or nuclei). Details on the rules of finding daughter cells can be found in [“Additional file 1”]. For each pair of daughter cells, the intensity of their interface is examined to determine whether the division has finished. The membrane-centered segmentation yields cell boundaries with the membrane signal or ridges in EDT. We calculated the average intensity of two cells’ interface to determine whether this interface located at ridges with a hole. If the interface includes a hole, the division is in process and two cells should be merged. The average intensity threshold is experimentally determined to be 40. Segmentation results after cell division correction is shown in Fig. 16c.

## Additional file

Additional file 1 Parameter settings of BCOMS and ACME in segmenting membrane images. It also includes steps on finding daughter cells in *Cell division revision* stage. (PDF 992 KB)

## Abbreviations

*C. elegans*: Caenorhabditis elegans
EDT: Euclidean distance transformation
MWMS: Membrane based Watershed Morphological Segmentation
