# Supplementary material for: 3DMMS: robust 3D Membrane Morphological Segmentation of C. elegans embryo
Source: BMC Bioinformatics. 2019 Apr 8;20:176. doi: 10.1186/s12859-019-2720-x (PMC6454620; doi:10.1186/s12859-019-2720-x)
Supplement: Supplementary file 1 — Parameter settings of BCOMS and ACME in segmenting membrane images. It also includes steps on finding daughter cells in Cell division revision stage. (PDF 992 KB) [file 12859_2019_2720_MOESM1_ESM.pdf]

1. Segmentation in RACE

1.1 Segmentation steps

Membrane stacks at 6 time points are resized into $205 \times 285 \times 134$ with uniform resolution on three directions. Nucleus stack is used as seeds in RACE. First, nucleus location information is extracted from *AceTree* and nucleus stack is constructed by initializing as zero matrix, and then setting corresponding nucleus pixels in nucleus stack as 255.

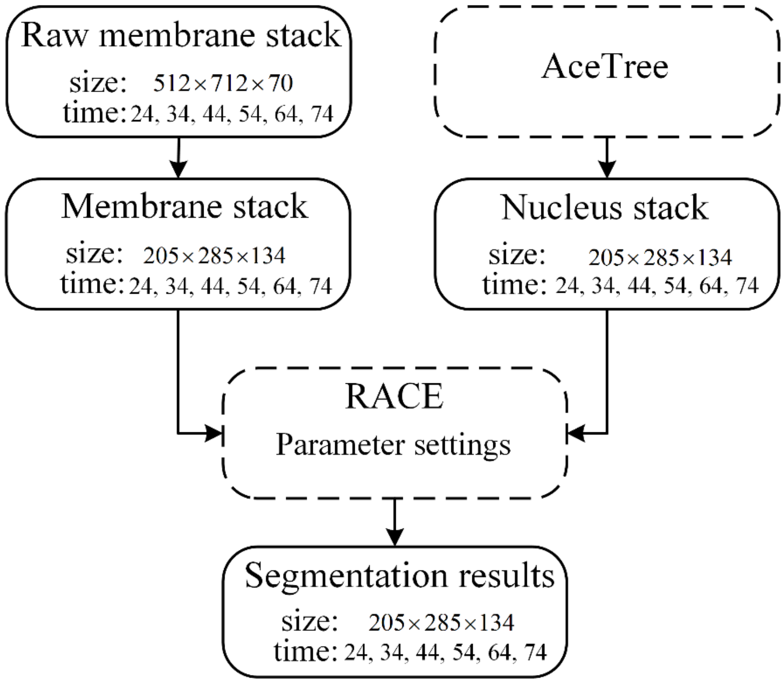

Fig.1 Steps of segmentation with RACE

1.2 Parameter settings

In order to get relatively optimal segmentation results, we test RACE under different parameters. Parameters that produces the best segmentation (used in comparison) are listed in Fig.2.

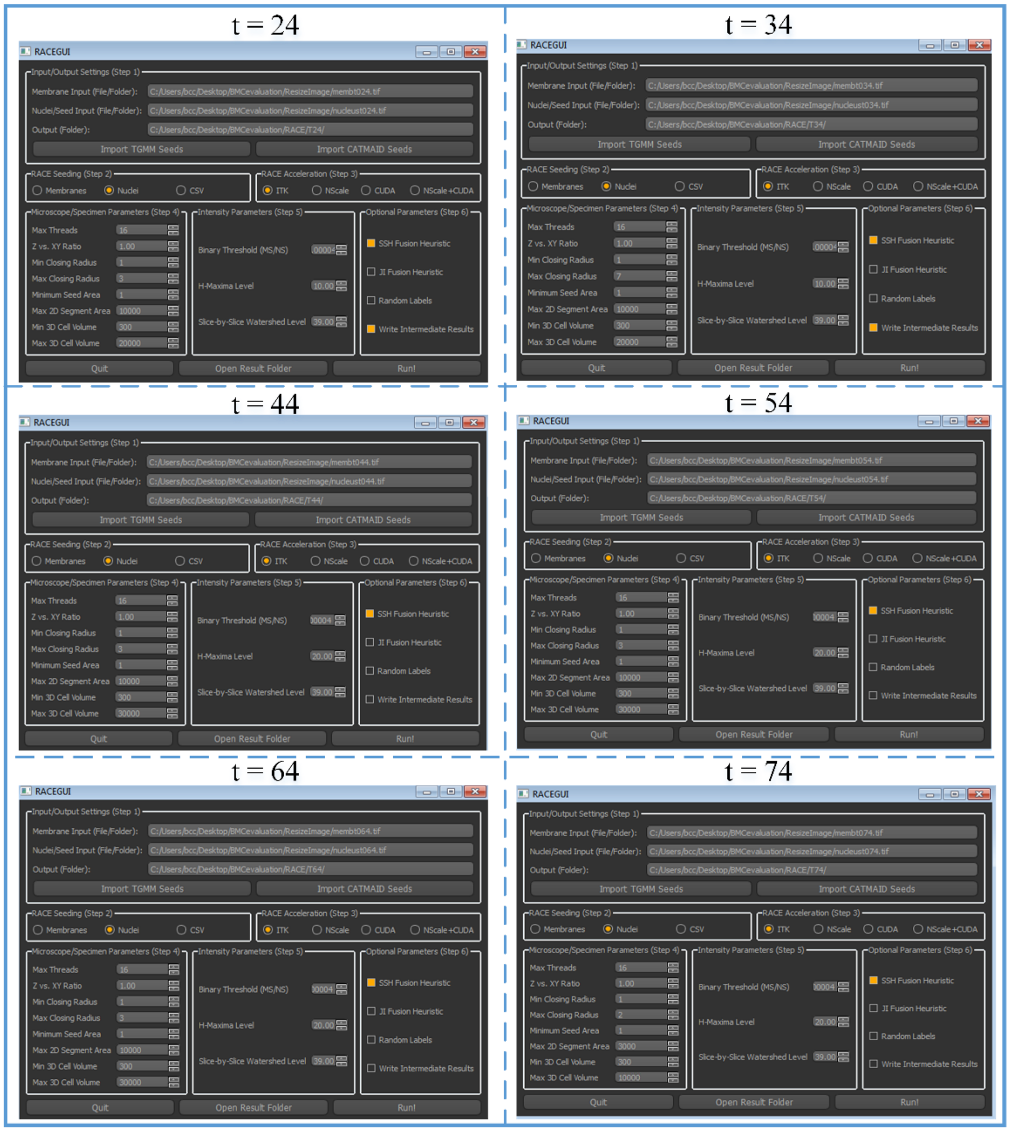

Fig.2 Parameters used in RACE

2. Segmentation in BCOMS

2.1 Segmentation steps

In BCOMS, time-lapse stacks need to be summed in *Embryonic region segmentation* stage. Three membrane stacks at 3 continuous time points (target *t* at the middle) are concatenated along the *z* direction as required in the manual of BCOMS. Nucleus stack are also constructed from *AceTree*, and concatenated to be seeds in watershed segmentation stage. Slices corresponding to the target *t* are stacked into the final results.

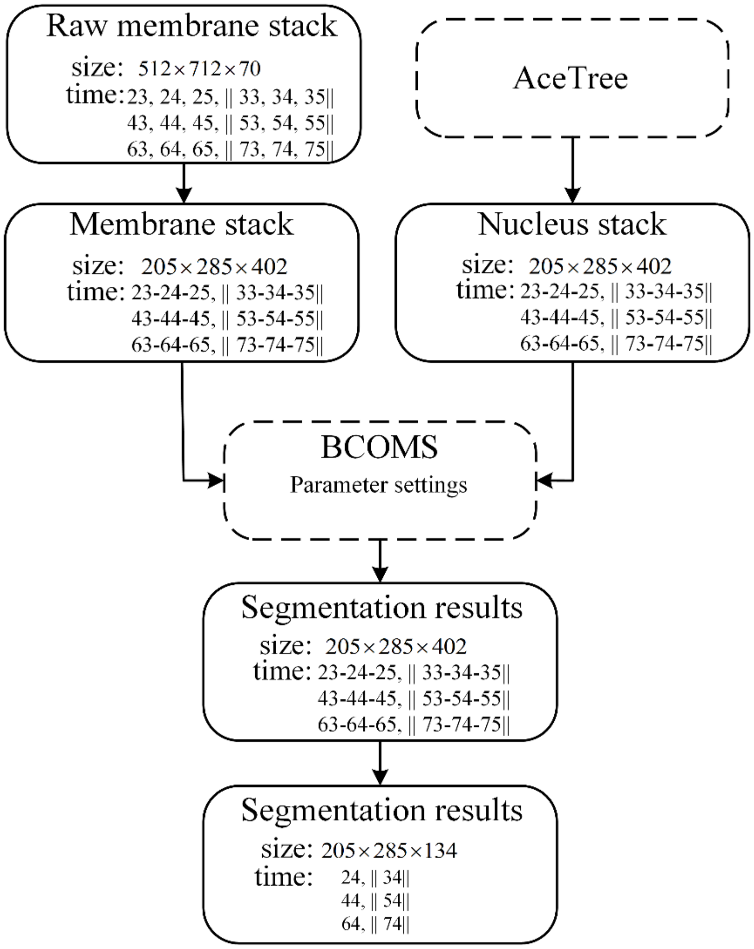

Fig.3 Schematic of segmentation with BCOMS

In BCOMS, membrane stacks at three series time points are concatenated vertically before being processed. For example, 23-24-25 represents that membrane stacks (after resample and resize) at $t = 23, 24, 25$ are combined, so one image to be processed includes $134 \times 3 = 402$ slices.

2.2 Parameter settings

Biological constrains reduce parameters to be tuned in BCOMS. Images are resampled before the input, so resolutions on *XY* and *Z* are set the same. This parameter setting is used for the segmentation at all time points.

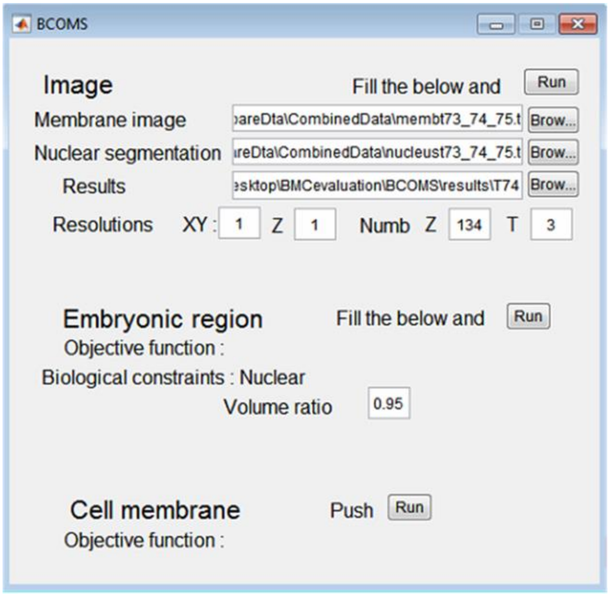

Fig.4 Parameters used in BCOMS

3. How to find daughter cells

Nuclei are named by *AceTree* based on the nomenclature described in [1]. Blast cells are given names comprised with numbers and uppercase. Cell's progenyis named by adding lower letters depicting the division direction with respect to the imaging coordinate system. Cells of the next generation are named in the same way by appending new letters. Therefore, we screen dividing cells by analyzing the name of each pair of nuclei. Nuclei are distinguished with different string variables S_i, where i: $1 < i < n$ is the serial number of nucleus. Then we construct division cell matrix DC_{nn} to find dividing cells.

$$DC(i, j) = \begin{cases} 1, \text{ if } S_i[1:end-1] = S_j[1:end-1] \\ 0, \text{ if } S_i[1:end-1] \neq S_j[1:end-1] \end{cases}$$

where *end* was the location of the last character in the string. If S_i, S_j are equal except the last character, the corresponding cells should have the same parent. Some special cases are treated individually, such as cell `P0' dividing into cell “AB” and “P1”.

Reference

[1]. Sulston J E , Schierenberg E , White J G , et al. The embryonic cell lineage of the nematode Caenorhabditis elegans[J]. Developmental Biology, 1983, 100(1):64-119.
